# Population structure analysis to explore genetic diversity and geographical distribution characteristics of wild tea plant in Guizhou Plateau

**DOI:** 10.1186/s12870-023-04239-2

**Published:** 2023-05-16

**Authors:** Limin He, Jing Luo, Suzhen Niu, Dingchen Bai, Yanjun Chen

**Affiliations:** 1 College of Tea Science / Institute of Agro-Bioengineering, Guizhou University, Guiyang, Guizhou Province 550025 People’s Republic of China; 2 Key Laboratory of Plant Resources Conservation and Germplasm Innovation in Mountainous Region, Guizhou University, Ministry of Education, Institute of Agro-Bioengineering, Guiyang, 550025 Guizhou Province People’s Republic of China

**Keywords:** Wild tea plant, Genetic diversity, Genotyping-by-sequencing, Guizhou Plateau, Population structure

## Abstract

**Background:**

Tea, the second largest consumer beverage in the world after water, is widely cultivated in tropical and subtropical areas. However, the effect of environmental factors on the distribution of wild tea plants is unclear.

**Results:**

A total of 159 wild tea plants were collected from different altitudes and geological types of the Guizhou Plateau. Using the genotyping-by-sequencing method, 98,241 high-quality single nucleotide polymorphisms were identified. Genetic diversity, population structure analysis, principal component analysis, phylogenetic analysis, and linkage disequilibrium were performed. The genetic diversity of the wild tea plant population from the Silicate Rock Classes of *Camellia gymnogyna* was higher than that from the Carbonate Rock Classes of *Camellia tachangensis*. In addition, the genetic diversity of wild tea plants from the second altitude gradient was significantly higher than that of wild tea plants from the third and first altitude gradients. Two inferred pure groups (GP01 and GP02) and one inferred admixture group (GP03) were identified by population structure analysis and were verified by principal component and phylogenetic analyses. The highest differentiation coefficients were determined for GP01 vs. GP02, while the lowest differentiation coefficients were determined for GP01 vs. GP03.

**Conclusions:**

This study revealed the genetic diversity and geographical distribution characteristics of wild tea plants in the Guizhou Plateau. There are significant differences in genetic diversity and evolutionary direction between *Camellia tachangensis* with Carbonate Rock Classes at the first altitude gradient and *Camellia gymnogyna* with Silicate Rock Classes at the third altitude gradient. Geological environment, soil mineral element content, soil pH, and altitude markedly contributed to the genetic differentiation between *Camellia tachangensis* and *Camellia gymnogyna*.

**Supplementary Information:**

The online version contains supplementary material available at 10.1186/s12870-023-04239-2.

## Background

Tea (*Camellia sinensis* (L.) O. Kuntze) is the second largest consumer beverage in the world after water, and is widely cultivated in tropical and subtropical areas [1, 2]. China is the origin of tea plants and has the broadest genetic variation globally, especially in the Guizhou Plateau. The altitude of Guizhou Plateau increases from approximately 200 m in the east to approximately 2,800 m in the west, and the plateau is predominantly composed of Carbonate Rock (CR) and Silicate Rock (SR) undergoing complicated and extensive folding, faulting, and stream erosion [3]. Altitude is known to play an important role in plant diversity [4, 5]. Further analysis of the effects of different geological characteristics, altitudes and soils formed at different altitudes on the genetic diversity of wild tea plants is of great significance to the genetic breeding of tea plants.

Molecular markers are essential for breeding major crops in current agriculture industry, and many molecular marker techniques have been developed and applied to the detection of genetic diversity and environmental adaptability of various crops [6–8]. Single nucleotide polymorphisms (SNPs) are the most effective marker system for determining DNA sequence variations and are responsible for specific traits or are used to track the evolutionary history of species. In addition, SNPs are the most common source of genetic variation in eukaryotes, and have become an important symbol of plant genetic research. The extensive SNP variation can be captured using genotyping-by-sequencing, which not only offers a cost-effective solution but also narrows the genotyping gap between local or specific populations [9, 10]. For example, a total of 3.6 million SNPs were identified in 517 rice varieties using next-generation sequencing, revealing the genetic basis of agronomic traits of rice to adapt to climate change [11]. Taranto et al. [12] identified 32,950 high-quality SNPs in *Capsicum annuum* germplasm through sequencing (genotyping-by-sequencing, GBS) and evaluated the genetic diversity in *C. annuum* germplasm. Meanwhile, Niu et al. [13] recognized 79,016 high-quality SNPs in 415 tea materials using the GBS method and explored the genetic diversity of tea germplasm resources in Guizhou Plateau.

Local adaptation is the basis of segregation of most phenotypic variations within species, with various environmental factors shaping the patterns of genetic variation and differentiation of wild germplasm resources [14]. Environmental factors, including light, temperature, water content, and especially altitude and geological rock, play an important role in plant genetic differentiation. Soils formed by different rocks exhibit significant differences in fertility, thus forming distinct suitable growth areas for plants [3]. Altitude also affects soil development owing to differences in soil moisture content, soil temperature, light, and other conditions. For instance, several granite endemic species in Western Australia have low diversity and high population differentiation [15]. In China, soil is a major evolutionary factor in the species richness model of subtropical plants (*Camelliaceae*) [16]. Differences in altitude cause differences of temperature, rainfall, and light in the region and subsequently the difference of population distribution and genetic diversity of plants. Such as, the overall evolution speed of azaleas growing at high altitudes is faster than that at low altitudes. Rhododendron growing at high altitude often resists the environmental heterogeneity caused by low temperature and altitude by improving its own carbohydrates, fatty acids, amino acids, and flavonoids. Rhododendron at low altitude usually adapts to low-altitude nematode, nitrogen, water deficiency, hypoxia, and acidic pH conditions by releasing the hormones salicylic acid (SA) and jasmonic acid (JA) [17, 18]. The Tibetan poplar (*Populus szechuanica var. tibetica* Schneid) was found to have the highest genetic diversity at low altitude and the lowest genetic diversity at high altitude [19]. Therefore, altitude and geological environment have an important impact on plant genetic diversity.

In our previous study, we investigated the agronomic characteristics, soil nutrient status, and ecological environment of wild tea plants in Guizhou, China and discovered significant differences in the altitude and ecological environment regarding growth of *Camellia tachangensis* and *Camellia gymnogyna*. *C. tachangensis* was predominantly distributed in the high-altitude carbonate rock area in the southwest of the Guizhou Plateau, while *C. gymnogyna* was predominantly distributed in the low-altitude silicate rock area in the north of the Plateau. *C. tachangensis* and *C. gymnogyna* have obvious differences in tree type, leaves, flowers, and fruits [20–22]. In the current study, we sought to further explore *C. tachangensis* and *C. gymnogyna* adapt to the evolution in different altitudes and geological environments of the Guizhou Plateau. Wild tea plants (159 in total) were collected from different altitudes and geological environments in Guizhou Plateau, and GBS was used to identify the significant SNPs of these plant materials. Subsequently, population structure analysis, principal component analysis (PCA), and phylogenetic analysis were conducted, and the 159 wild tea plants were divided into three groups (GP01, GP02, and GP03). In addition, linkage disequilibrium (LD), genetic diversity, and soil nutrient characteristics of the different populations were analyzed. Findings from the study provide valuable information for future tea breeding and genetic analysis.

## Results

### Sequencing and variant discovery

**Fig. 1:**
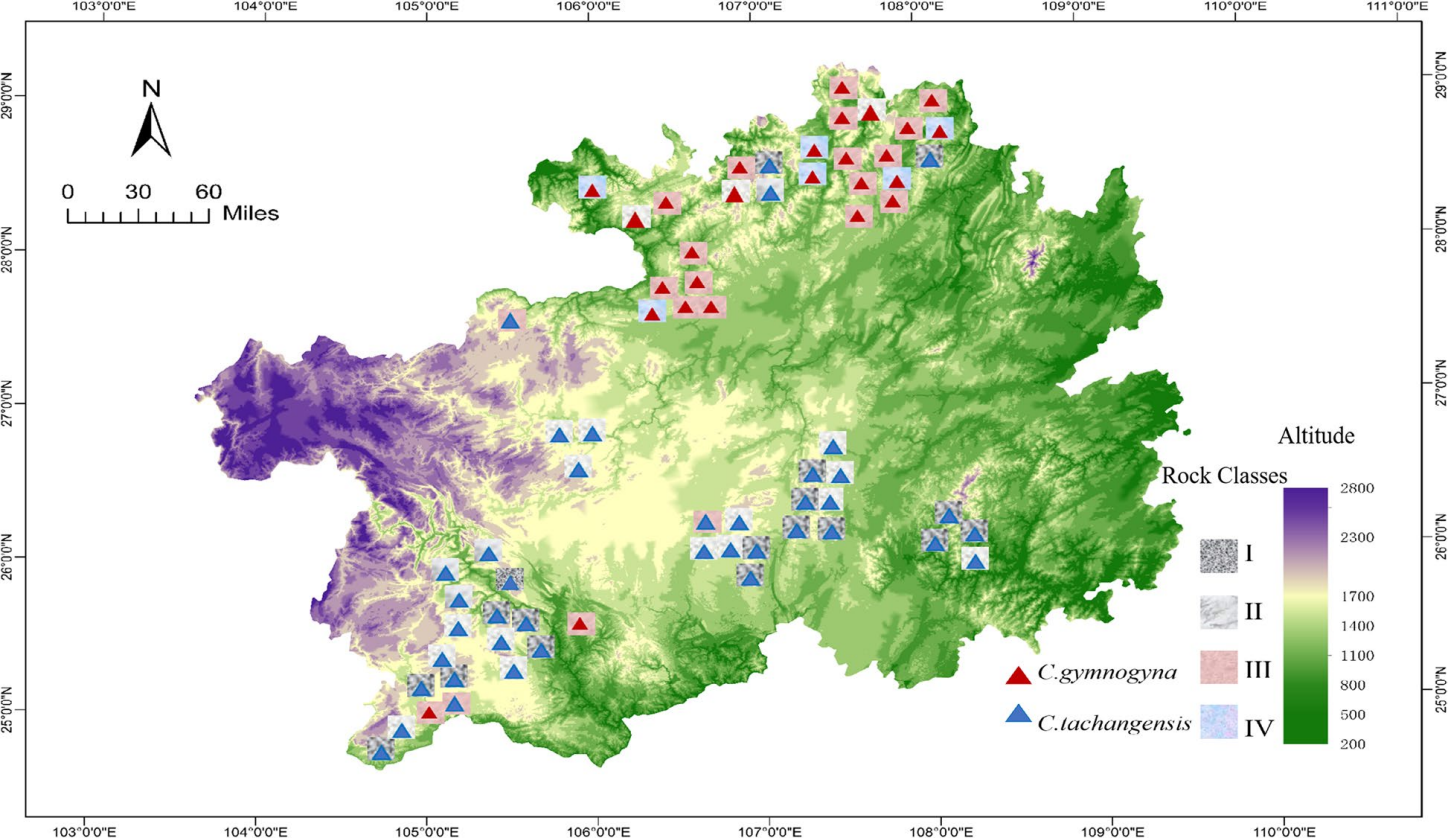
Geographic distribution of 159 tea accessions. Note: Geographic distribution of accessions are represented by triangles on the altitude map of Guizhou. The red triangle represents *C. gymnogyna* and the blue triangle represents *C. tachangensis*. I Dolomite sub-suitable area, II Dolomitic limestone suitable area, III Clastic rock most suitable area, IV Purple clastic rock suitable area. The altitude is rising from pure green to purple

**Fig. 2:**
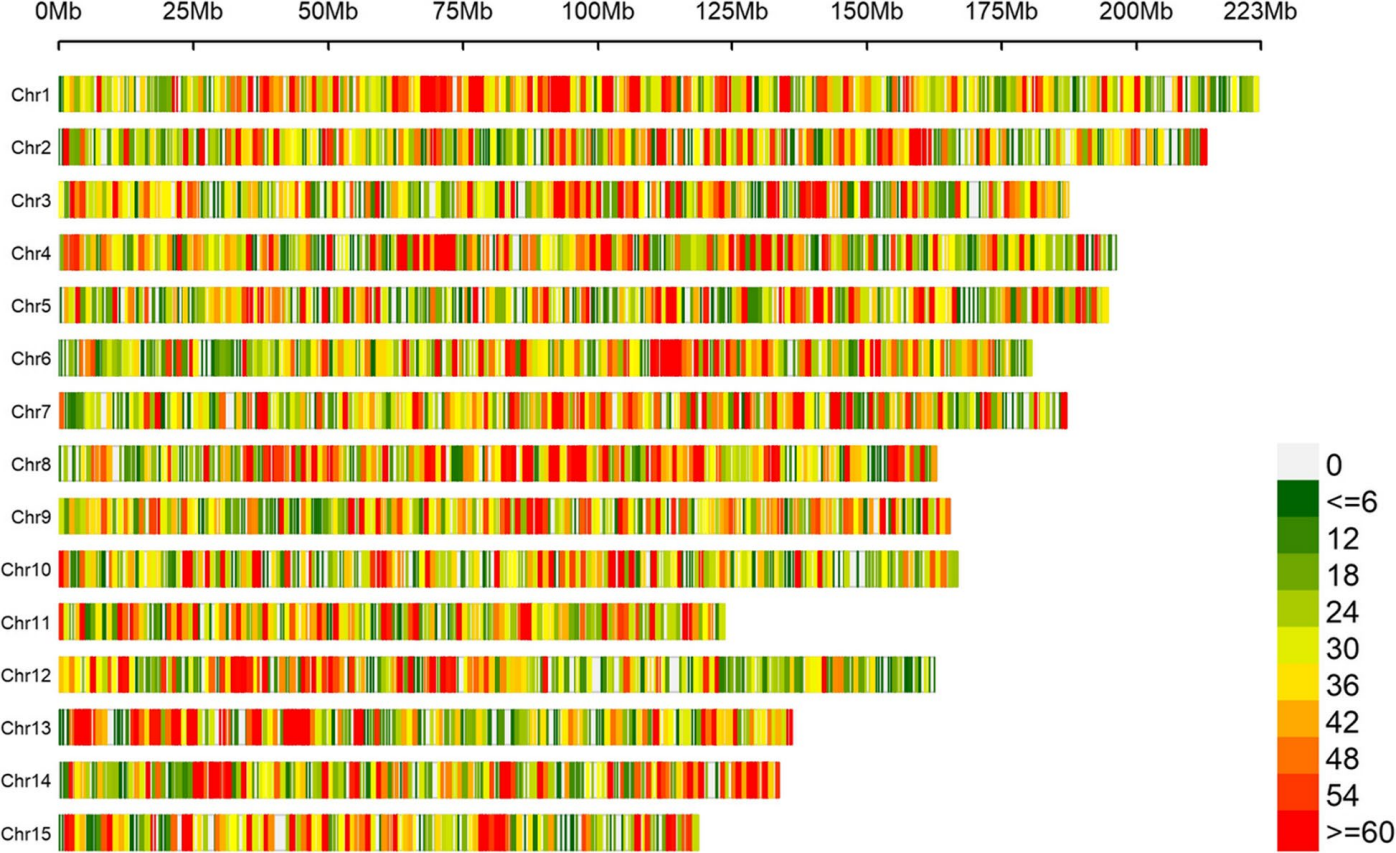
Density of SNPs of 159 accessions. Distribution map of SNPs on 15 chromosomes graph

To explore the genetic variation of the wild tea plant population of Guizhou Plateau, 159 wild tea plants, comprising 101 *C. tachangensis* plants and 58 *C. gymnogyna* plants, were collected based on the different altitudes and geological types of Guizhou Plateau (Additional file 1: Table S1). The geographical characteristics and distribution of the 159 wild tea plants in Guizhou Plateau are shown in Fig. 1. Subsequently, 162.9 Gb clean sequencing data with an average of 1.02 Gb per accession were obtained (Additional file 1: Table S2), which were further mapped to the reference genome of tea (http://tpia.tea.plant.org/). SNPs were then detected and genotyped by GATK (version 3.7.0) based on the reference genome [23]. In total, 29,393,327 SNPs were identified and the heterozygosity value was calculated. The average heterozygosity rate per accession was 8.16% (Additional file 1: Table S3). The filter left 98,241 high-quality SNPs, which were unevenly distributed on 15 chromosomes. The smallest and largest SNP density was detected on chromosome 15 and chromosome 1, respectively (Fig. 2; Additional file 1: Table S4), while the average number of SNPs per chromosome was 9,162. Analysis of nucleotide substitutions showed that the 98,241 SNPs were classified into two types: transition and transversion. There were 911,455 (78.10%) transitions and 255,518 (21.90%) transversions. The substitution frequencies were 79,404 (6.80%) A/T, 63,029 (5.40%) A/C, 66,018 (5.66%) G/T, 47,067 (4.03%) C/G, 456,661 (39.13%) C/T, and 454,794 (38.97%) A/G. The transition to transversion ratio was 3.57 (Table 1).

**Table 1:** Percentage of transition and transversion SNPs identified using genotyping-by-sequencing

	Transitions		Transversions			
	AG	CT	AT	AC	CG	GT
Numbers of allelic sites	454,794	456,661	79,404	63,029	47,067	66,018
Percentage of allelic sites	38.97%	39.13%	6.80%	5.40%	4.03%	5.66%
Total (Percentage)	911,455 (78.10%)		255,518 (21.90%)			

### Genetic diversity estimation

Nucleotide diversity (*Pi*), observed heterozygosity (*Ho*), minor allele frequency (*MAF*), and inbreeding coefficient (*Fis*) are recognized indicators of genetic diversity. In this study, *Pi*, *Ho*, *MAF*, and *Fis* of the 159 wild tea plants were 0.230, 0.085, 0.149, and 0.644, respectively (Table 2). Further analysis of the genetic diversity of tea plant populations from two different species showed that *Ho* and *MAF* of the *C. gymnogyna* tea plant population were significantly higher than those of the *C. tachangensis* population. *Fis* was higher for the *C. tachangensis* population compared with the *C. gymnogyna* population. Analysis of the genetic diversity of tea plant populations from three different altitude gradients revealed that *Pi*, *Ho*, and *MAF* of the tea plant population from H2 were significantly higher compared with those from H3 and H1, and *Pi*, *Ho*, and *MAF* of the tea plant population from H3 was significantly higher than that of the population from H1. Comparison of the genetic diversity of tea plant populations in the CR and SR Classes of Guizhou Plateau revealed that *Ho* and *MAF* of the tea plant population in SR were significantly higher compared with those for the tea plant population in CR (Table 2). Among the four geological areas, *Pi* and *MAF* of the tea plant population in I were significantly higher compared with those in III, IV, and II (Table 2).

**Table 2:** Genetic diversity parameters of 159 wild tea germplasm resources in Guizhou Plateau

Type		Number	Ho	Fis	Tajima’s D	MAF	Pi
Species	*C. tachangensis*	101	0.080b	0.652a	0.650	0.143b	0.220a
	*C. gymnogyna*	58	0.087a	0.577b	0.721	0.148a	0.219a
Altitude	H1	39	0.079c	0.567a	0.718	0.126c	0.185c
	H2	65	0.088a	0.625a	0.543	0.148a	0.227a
	H3	55	0.086b	0.620a	0.627	0.144b	0.215b
Classes	CR	92	0.083b	0.628a	0.585	0.141b	0.217a
	SR	67	0.088a	0.604a	0.736	0.144a	0.215a
Areas	I	48	0.086b	0.624a	0.404	0.145a	0.221a
	II	44	0.080c	0.602a	0.303	0.132c	0.198c
	III	46	0.089a	0.603a	0.591	0.143b	0.214b
	IV	21	0.088a	0.586a	0.539	0.143b	0.213b
All	all	159	0.085	0.644	0.941	0.149	0.230

*Pi* nucleotide diversity, *Ho* observed heterozygosity, *MAF* minor allele frequency, *Fis* inbreeding coefficient. In the same type and line, the different letters indicate a significant difference at *P* = 0.05 level by *t*-test. Species contain *C. tachangensis* and *C. gymnogyna*. Altitude contains H1, H2 and H3, which are the first (> 1400 m), second (1400–1100 m), and third (< 1100 m) altitude gradients, respectively. Classes contain CR (Carbonate Rock) and SR (Silicate Rock). Areas contain I Dolomite sub-suitable area, II Dolomitic limestone suitable area, III Clastic rock most suitable area, and IV Purple clastic rock suitable area

Positive values of Tajima’s D arise from an excess of intermediate frequency alleles and can result from population bottlenecks, structure, and/or balancing selection [24]. In this study, a positive value of Tajima’s D was detected in the wild tea plant population, suggesting that the wild tea plant population underwent population bottlenecks and/or balancing selection (Table 2). *Fst* (differentiation coefficient) is used as a measure of population structure; *Fst* values of 0–0.05 and 0.05–0.15 indicate little and moderate divergence, respectively [25, 26]. In this study, the *Fst* value of *C. gymnogyna* and *C. tachangensis* was 0.075, suggesting that the moderate divergence occurred in the wild tea plant population between *C. gymnogyna* and *C. tachangensis* (Table 3). In addition, the *Fst* values of geological areas I and III, I and IV, II and III, and II and IV were 0.056, 0.063, 0.126, and 0.145, respectively, suggesting that the moderate divergence occurred in the wild tea plant population between I and III between I and IV, between II and III, and between II and IV. The *Fst* values of geological areas I and II, III and IV were 0.036 and 0.003, respectively, suggesting that little divergence occurred in the wild tea plant population between I and II, and between III and IV. The highest pairwise genetic distance (*GD*) was for II vs*.* IV, while the lowest pairwise *GD* was for III vs*.* IV (Table 4). The *Fst* values in H1 vs. H2 were 0.032, indicating that little divergence occurred in H1 vs. H2. Meanwhile, the *Fst* values in H2 vs. H3 and H1 vs*.* H3 were 0.071 and 0.149, respectively, suggesting that moderate divergence occurred in H2 vs. H3 and in H1 vs*.* H3. The highest pairwise *GD* was for H1 vs. H3, while the lowest pairwise *GD* was for H1 vs. H2 (Table 5).

**Table 3:** *Fst* and pairwise genetic distance (*GD*) of *C. tachangensis* and *C. gymnogyna* populations

	*C. tachangensis*	*C. gymnogyna*
*C. tachangensis*		0.234
*C. gymnogyna*	0.075	

The bottom left is the value of pairwise genetic differentiation coefficients (*Fst*); the upper right is the value of pairwise genetic distance (*GD*)

**Table 4:** *Fst* and pairwise genetic distance (*GD*) among four geologically suitable area populations

	I	II	III	IV
I		0.169	0.182	0.187
II	0.036e		0.202	0.210
III	0.056d	0.126b		0.159
IV	0.063c	0.145a	0.003f	

The bottom left is the value of pairwise genetic differentiation coefficients (*Fst*); the upper right is the value of pairwise genetic distance (*GD*). Different letters indicate a significant difference at *P* = 0.05 level by *t*-test. I Dolomite sub-suitable area, II Dolomitic limestone suitable area, III Clastic rock most suitable area, IV Purple clastic rock suitable area

**Table 5:** *Fst* and pairwise genetic distance (*GD*) of three altitude gradient populations

	H1	H2	H3
H1		0.176	0.212
H2	0.032c		0.180
H3	0.149a	0.071b	

The bottom left is the value of pairwise genetic differentiation coefficients (*Fst*); the upper right is the value of pairwise genetic distance (*GD*). Different letters indicate a significant difference at *P* = 0.05 level by *t*-test. H1 the first altitude gradient (> 1400 m); H2 the second altitude gradient (1400–1100 m); H3 the third altitude gradient (< 1100 m)

### Population structure, PCA, and phylogenetic analysis

**Fig. 3:**
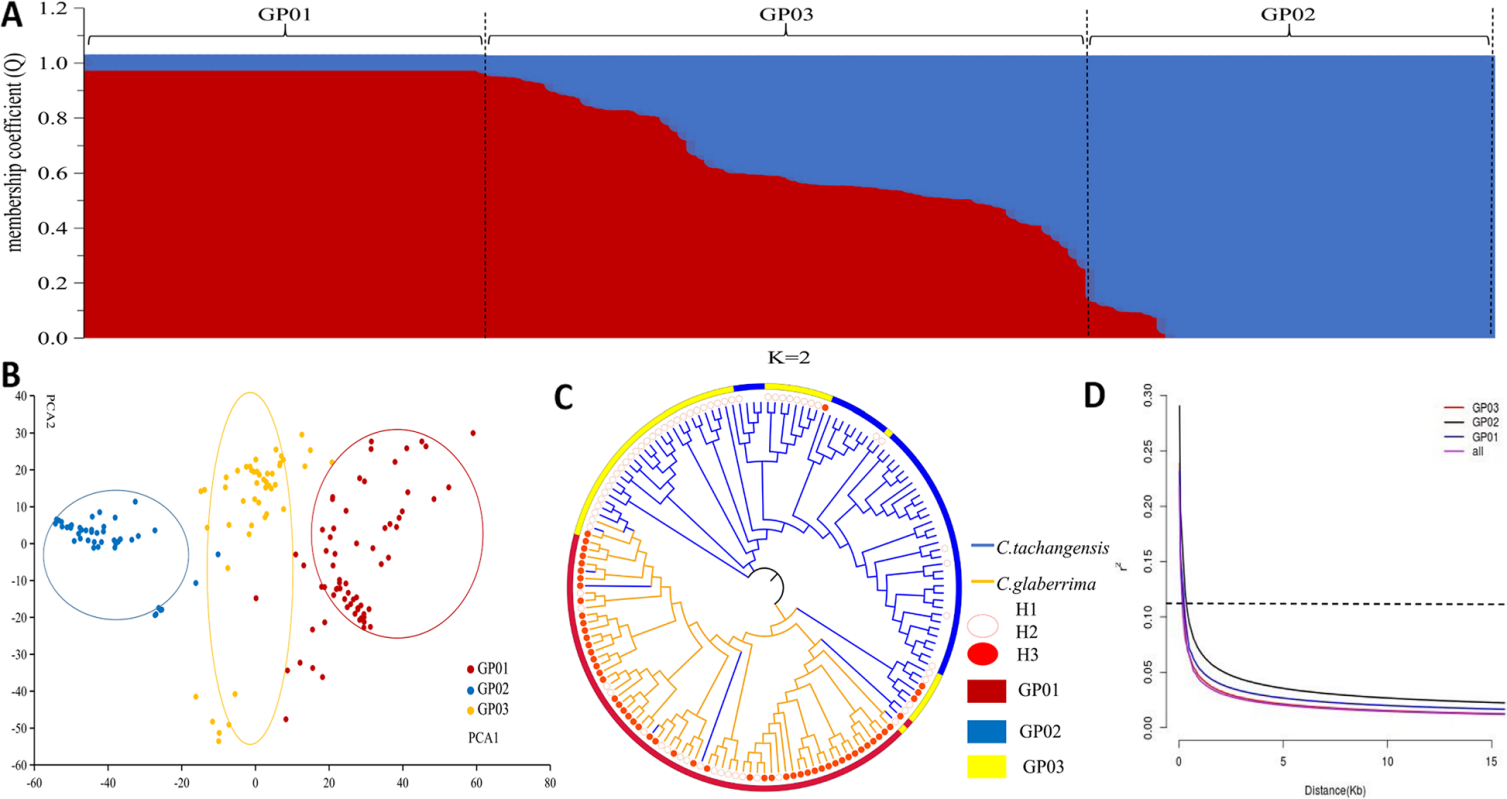
Population structure and LD of 159 accessions. **A** Inferred population structure of 159 accessions. Bar plot of individual membership coefficients for the genetic clusters was inferred using ADMIXTURE (K = 2) based on 98,241 SNPs. Individual membership coefficients (Q) were sorted within each cluster. GP01 and GP02 are shown in red and blue, respectively. **B** Principal component analysis (PCA). The three PCA scatter diagram was made by the first and second principal components. Three inferred populations were identified in ADMIXTURE, GP01 is shown in red, GP02 in blue, and GP03 in yellow. **C** ML tree comparing the classification of inferred populations, Species *C. tachangensis* and *C. gymnogyna*, altitude: H1, H2, and H3 corresponding to the first (> 1400 m), second (1400–1100 m), and third (< 1100 m) altitude gradient, respectively. **D** LD decay plot of 159 accessions and three inferred populations

To further explore the relationship of the 159 wild tea plant populations, the 98,241 high-quality SNPs were used to perform population structure analysis, PCA, and phylogenetic analysis. Dynamic changes in population structure were further estimated under different K values (K = 1–9). Analysis of cross-validation error (CV error) revealed that the CV error changed little as K increased from 1 to 9, and CV error was minimal at K = 2 (Additional file 2: Fig. S2). Accessions with membership coefficients > 0.80 were assigned to the corresponding pure groups, while those with coefficients < 0.80 were assigned to the admixture group (Additional file 1: Table S1) [11]. This resulted in the 159 wild tea plants being further classified into three population groups—two ancestral groups and one admixture group (Additional file 1: Table S1). The first ancestral group (referred to as ‘III and H3 of *C. gymnogyna* (III H3 *C. gymnogyna*)’ or ‘GP01’ from now on) contained 67 accessions, including 55 (82.0%) *C. gymnogyna* and 12 (18.0%) *C. tachangensis*, which all came from the SR Classes (46 (68.6%) tea accessions from area III, and 21 (31.4%) tea plants from area IV) (Additional file 1: Table S5), and these accessions were also divided into two clusters based on altitude (17 (25.3%) tea accessions from H2 and 50 (74.7%) tea accessions from H3) (Additional file 1: Table S6). The second ancestral group (referred to as ‘II and H1 of *C. tachangensis*’ (II H1 *C. tachangensis*) or ‘GP02’ from now on) contained 45 *C. tachangensis*, which came from the CR Classes (including 11 (24.4%) accessions from area I and 34 (75.6%) accessions from area II), with 39 (86.6%) plants from H1 and 6 (13.4%) plants from H2. The remaining 47 accessions formed an admixed group (referred to as ‘I and H2 of *C. tachangensis*’ (I H2 *C. tachangensis*) or ‘GP03’ from now on) that, comprised 44 (93.6%) *C. tachangensis* and 3 (6.4%) *C. gymnogyna*, with 37 (78.7%) plants collected from area I and 10 (21.3%) individuals collected from area II, and 42 (89.3%) and 5 (10.7%) of the accessions were distributed in H2 and H3, respectively (Additional file 1: Table S6). To identify potential population stratification, PCA and a maximum-likelihood phylogenetic tree (ML tree) were used to investigate dendrogram relationships among the 159 wild tea accessions using the 98,241 SNPs (Fig. 3C and Additional file 2: Fig S3). PCA and the ML tree revealed three major clusters corresponding to groups GP01, GP02, and GP03 from the population structure analysis, which further confirmed the accuracy of the population grouping (Fig. 3A and B).

### LD analysis

LD analysis is used to clarify domestication and breeding history. LD was estimated for the population of 159 wild tea accessions by using 29,393,327 non-pruned LD SNPs. The LD (r^2^) rapidly decayed with increasing physical distance. The maximum r^2^ value was 0.226 for the LD decay of all 159 accessions. As r^2^ decayed to half maximum (0.113), the corresponding physical distance was 0.116 Kb (Fig. 3D). Moreover, the slowest LD was identified in GP02, with LD decay of 0.260 Kb at r^2^ = 0.113. The physical distance for GP01 was 0.135 Kb at r^2^ = 0.113. The fastest LD decay was for GP03 with 0.120 Kb at r^2^ = 0.113.

### Genetic differentiation analysis of the inferred populations

**Fig. 4:**
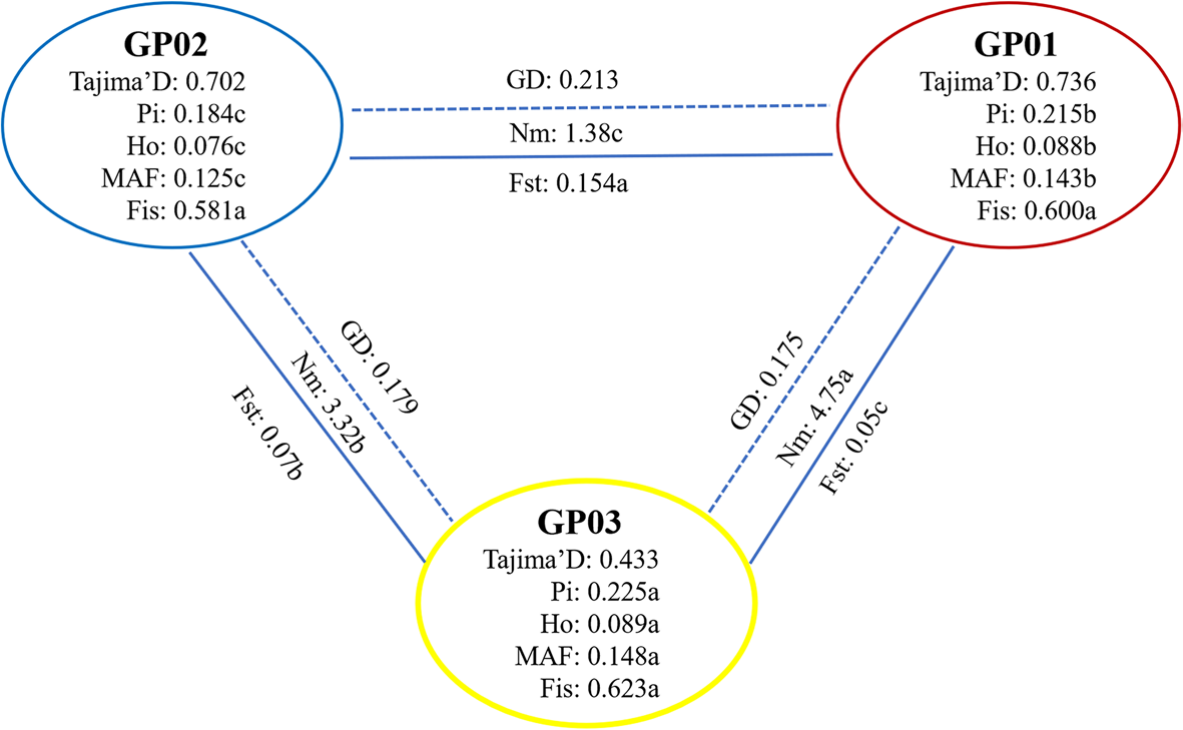
Genetic diversity of three inferred populations of 159 accessions Note: *Pi,* nucleotide diversity; *Ho,* observed heterozygosity; *MAF,* minor allele frequency; *Fis,* inbreeding coefficient; *GD,* genetic distance; *Fst,* differentiation coefficient; *Nm,* gene flow. Different letters indicate a significant difference in *p* = 0.05 levels by *t*-test. GP01 and GP02 are pure groups and GP03 is the admixture group base on ADMIXTURE software at K = 2

To further investigate the genetic diversity of the inferred populations, the Tajima’s D, *Pi*, *Ho*, and *MAF* of GP01, GP02 and GP03 were calculated. *Ho*, *Pi*, and *MAF* were significantly higher for GP03 compared with those of GP01 and GP02, and *Pi*, *Ho*, and *MAF* were significantly higher for GP01 than for GP02 (Fig. 4). The three groups had positive Tajima’s D values, indicating that all three groups underwent population bottlenecks, and/or balancing selection (Fig. 4).

Previous studies showed that *Fst* in the range of 0.00 to 0.05 indicate little divergence, *Fst* in the range of 0.05 to 0.15 indicate moderate divergence and 0.15 to 0.25 indicate large divergence [26, 27]. The pairwise *Fst* was analyzed among three inferred groups (Fig. 4). The *Fst* between GP01 and GP03, and GP02 and GP03 was 0.05 and 0.07, respectively, indicating moderate divergence, while the mean *Fst* between GP01 and GP02 was 0.154, indicating large divergence. The highest level of difference was detected between GP01 and GP02, and the lowest level of difference was detected between GP01 and GP03, while an intermediate differentiation was observed between GP02 and GP03 (Fig. 4). A lowest *GD* was detected on GP01 vs GP03 and GP02 vs GP03, while the highest *GD* was detected on GP01 vs GP02. The higher gene flow (*Nm*) was detected for GP01 vs GP03 and GP02 vs GP03, while the lowest *Nm* was detected for GP01 vs GP02. Therefore, there are more gene exchanges between GP01 and GP03, GP02 and GP03 and less gene exchange between GP01 and GP02 (Fig. 4).

In the analysis of molecular variance (AMOVA), no variants were detected in other populations such as GP01, GP02, and GP03, indicating that these populations have the same genetic basis. Most of the variants were detected within population (91.5%) and 8.5% were found among population (Table 6).

**Table 6:** AMOVA for different populations in wild tea plant

source of variation	d. f	sum of squares	variance components	percentage of variation	Fixation indexes	*p*-Values
GP01, GP02 and GP03 groups						
Among populations	2	8687.6	39.47	8.5	*Fst* = 0.085	< 0.0001
Within populations	303	128,567.6	424.31	91.5		< 0.0001
Total	305	137,255.2	460.78			
*C. tachangensis* and *C. gymnogyna* groups						
Among populations	1	4876.9	30.37	6.8	*Fst* = 0.068	< 0.001
Within populations	314	130,946.4	417.03	93.2		< 0.001
Total	315	137,354.6	477.40			
H1, H2 and H3 groups						
Among populations	2	6532.1	29.42	6.8	*Fst* = 0.068	< 0.001
Within populations	317	128,354.6	404.90	93.2		< 0.001
Total	319	134,886.7	434.33			
I, II, III and IV groups						
Among populations	2	5400.5	21.98	5.1	*Fst* = 0.05	< 0.001
Within populations	315	129,574.8	411.35	94.9		< 0.001
Total	317	134,975.3	433.33			

H1 the first altitude gradient (> 1400 m), H2 the second altitude gradient (1400–1100 m), H3 the third altitude gradient (< 1100 m). I Dolomite sub-suitable area, II Dolomitic limestone suitable area, III Clastic rock most suitable area, IV Purple clastic rock suitable area

### Characteristics of soil nutrients and altitude factors in different populations

Statistical analyses were conducted on soil nutrient content, pH, mean annual temperature (MAT) and mean annual rainfall (MAR) of different species, altitude, rock types, and groups (Table 7). The analysis of variance showed that soil organic matter (SOM), alkaline nitrogen (AN), total phosphorus (TP), available potassium (AK), exchangeable magnesium (Mg), exchangeable calcium (Ca) content, pH and MAR of *C. tachangensis* were significantly higher compared with those of *C. gymnogyna*, while the exchangeable aluminum (Al) content and MAT of *C. gymnogyna* were significantly higher than those of *C. tachangensis*. The total nitrogen (TN), exchangeable Ca content and pH of H1 were significantly higher than those of H2 and H3, while the exchangeable Al content and MAT were significantly lower compared with those of H2 and H3. The SOM, AN, TP, AK, exchangeable Mg, pH, and MAR of H1 and H2 were significantly higher than those of H3. The SOM, TN, AN, TP, AK, exchangeable Mg, exchangeable Ca, pH, and MAR of CR were significantly higher compared with those of SR, while the contents of AP, exchangeable Al and MAT were significantly lower compared with those of SR. The content of exchangeable Al in soil I was significantly lower than that in soils II, III, and IV, and the content of AK was significantly higher than that in soils II, III, and IV. The SOM, AN, TP, exchangeable Mg, exchangeable Ca and MAR in soils I and II were significantly higher compared with those in soils III and IV. In addition, the SOM, TN and pH of GP02 were significantly higher than those of GP03 and GP01, and the SOM, TN, AN, TP, exchangeable Mg, exchangeable Ca content, pH, and MAR of GP02 and GP03 were significantly higher than those of GP01.

**Table 7:** ANOVA for soil nutrients and altitude factors in different groups

Type		Number	SOM	TN	AN	TP	AP	AK	Exchangeable Al	Exchangeable Mg	Exchangeable Ca	pH	MAT	MAR
Species	*C. tachangensis*	101	49.15a	2.25a	129.14a	1.86a	5.23a	64.37a	0.72b	1.51a	5.34a	5.14a	15.3b	1326a
	*C. gymnogyna*	58	38.06b	1.95a	110.81b	0.44b	8.35a	57.30b	0.90a	0.70b	4.79b	4.52b	15.8a	1194b
Altitude	H1	39	58.28a	2.58a	128.09a	1.75a	5.75b	63.54a	0.61c	1.49a	5.62a	5.25a	14.3b	1321a
	H2	65	44.52a	2.09b	130.57a	1.59a	5.99ab	65.52a	0.75b	1.31a	5.41b	4.94b	15.8a	1286a
	H3	55	36.46b	1.88b	108.86c	0.77b	7.25a	56.15b	0.95a	0.92b	4.48b	4.65c	15.9a	1238b
Classes	CR	92	50.92a	2.31a	131.34a	1.76a	5.28b	64.28a	0.66b	1.49a	5.52a	5.12a	15.2b	1319a
	SR	67	37.12b	1.89b	110.25b	0.77b	7.86a	58.37b	0.96a	0.85b	4.62b	4.63b	15.8a	1221b
Areas	I	48	46.61a	2.16ab	133.48a	1.62a	5.08b	67.07a	0.58b	1.41a	5.35a	5.09b	15.8a	1320a
	II	44	57.81a	2.49a	129.01a	1.92a	5.50b	61.24b	0.74a	1.56a	5.71a	5.15a	14.6b	1318a
	III	46	36.20b	1.89b	112.00b	1.03b	7.74a	57.74b	0.99a	0.95b	4.59b	4.72c	15.8a	1240b
	IV	21	39.12b	1.89b	106.41b	0.19c	8.15a	59.75b	0.88a	0.62b	4.67b	4.42d	15.8a	1179b
Groups	GP01	67	37.12c	1.89c	110.25c	0.77b	7.86a	58.37a	0.96a	0.85b	4.62b	4.63c	15.81a	1221b
	GP02	45	55.26a	2.49a	128.47ab	1.77a	5.65b	63.46a	0.64b	1.52a	5.69a	5.24a	14.46b	1324a
	GP03	47	46.77b	2.15b	134.08a	1.75a	4.93b	65.07a	0.68b	1.45a	5.36a	5.01b	15.97a	1315a
All	all	159	45.11	2.14	122.45	1.34	6.37	61.79	0.79	1.21	5.14	4.92	15.5	1278

SOM soil organic matter, TN total nitrogen, AN alkaline nitrogen, TP total phosphorous, AP available phosphorous, AK available potassium, MAT,mean annual temperature, MAR mean annual rainfall. In the same type and line, the different letters indicate a significant difference at *P* = 0.05 level by t-test. Species contain *C. tachangensis* and *C. gymnogyna*. Altitude contains H1, H2, and H2, which are the first (> 1400 m), second (1400–1100 m), and third (< 1100 m) altitude gradient, respectively. Classes contain CR (Carbonate Rock) and SR (Silicate Rock). Areas contain I Dolomite sub-suitable area, II Dolomitic limestone suitable area, III Clastic rock most suitable area, and IV Purple clastic rock suitable area

## Discussion

Prior research has highlighted the influence of environmental factors on plant genetic differentiation [28–30]. In the current investigation, factors such as altitude and geological type emerge as key drivers in the evolutionary process of wild tea plants.

### Genetic diversity

In this study, 29,393,327 initial SNPs and 98,241 high-quality SNPs after applying filtering criteria were used to detect the genetic diversity of 159 wild tea plants from the Guizhou Plateau. In our previous report, 1,001,372 initial SNPs and 79,016 high-quality SNPs were obtained from 415 tea plants, which are lower than the high-quality SNPs in our material study under the same filtering conditions [13]. This demonstrates that it is feasible to analyze the genetic diversity and genetic differentiation of 159 wild tea plants with 98,241 high-quality SNPs by the GBS method.

Life-history or geographic traits play an important role in genetic diversity [31]. In general, less genetic diversity exists in an endemic species that is not widely distributed compared with that found in a widespread species [32], usually because their population numbers are limited, and as they are isolated from other populations they adapt to their particular habitat [33]. In this study, the *Ho* and *MAF* of the *C. gymnogyna* population were significantly higher than that of the *C. tachangensis* population, while *Fis* of the *C. tachangensis* population was significantly higher than that for *C. gymnogyn*, suggesting that there was rich genetic diversity in the *C. gymnogyn* population. The SOM, AN, TP, AK, exchangeable Mg and Ca contents, and pH of the *C. tachangensis* population were significantly higher compared with those of the *C. gymnogyn* population. Previous studies have revealed that *C. gymnogyn* may have evolved from *C. tachangensis* [34]. *C. tachangensis* is relatively primitive, which may adapt to the natural environment with high soil nutrient content, pH and low temperature in Guizhou Plateau for a long time. *C. tachangensis* has conducted frequent inbreeding, reduced communication with other tea plants, and has low genetic diversity.

Earlier research has uncovered that a suitable environment will promote gene exchange between plants and increase the genetic diversity of plants. The favorable and mild environmental conditions at mid-altitude mean plant populations at this altitude exhibit greater diversity compared with those at low and high altitudes [19, 35, 36]. In this study, genetic diversity was significantly higher for the wild tea population at H2 than for the wild tea populations at H3 and H1. In addition, the genetic diversity was significantly higher for the wild tea populations in the SR Classes compared with those in the CR Classes. Among the four suitable areas, the genetic diversity of wild tea plant populations was significantly higher in area I compared with those in areas III, IV, and II (Table 2). The MAT of H1 was significantly lower than that of H2 and H3, and the SOM, AN, TP and AK contents of H2 were significantly higher than those of H3. The SOM, TN, AN, TP, AK, exchangeable Mg and Ca contents, and pH of CR were significantly higher than those of SR. In addition, the exchangeable Al content of area I was significantly lower compared with that in areas, II, III, and IV. A reasonable explanation for the results of this study is that the low MAT in the H1 areas delayed the flowering time of wild tea plants, hindered the natural hybridization between tea plants, and ultimately reduced the genetic diversity of wild tea plants [37]. In contrast, H2 areas with a suitable temperature and high soil nutrient content promote the natural hybridization of wild tea plants, which accounts for wild tea plants in H2 area exhibiting the highest genetic diversity [38]. The CR region has suitable soil nutrients, but it is possible that wild tea plants have reduced genetic diversity due to long-term adaptation to local suitable environments. Studies have shown that species can adapt to the selection pressure on their own growth by improving their genetic diversity [39, 40]. On the other hand, unfavorable soil or environment in general may kill off a lot of plants in the populations thus reducing the genetic diversity. Our results show that the genetic diversity of the wild tea plant population in I areas is higher than that in III, IV and II areas, and the exchangeable Al in I areas is significantly lower than that in III, IV, and II areas, indicating that the high diversity of wild tea plants in I areas may be more affected by the selection pressure of the low Al soil environment.

In addition, it has been shown that positive Tajima’s D indicated population bottlenecks and/or balancing selection [24]. In our study, positive Tajima’s D values were observed in all populations, suggesting that population bottlenecks and/or balancing selection occurred in wild tea plant populations (Table 2). *Fst* has been widely used as a measure of population structure. The *Fst* values for wild tea plant populations of *C. tachangensis* and *C. gymnogyn*, H1 and H3, H2 and H3, areas II and III, and areas II and IV were 0.05–0.15, indicating that moderate divergence occurred in wild tea plant populations between *C. tachangensis* and *C. gymnogyn*, between H1 and H3, between H2 and H3, between areas II and III, and between areas II and IV [25, 26].

### Population structure

Environmental factors, including geological environment, altitude, temperature, rainfall, and light, significantly affect the growth of tea plants. In particular, altitude and geological environment played important roles in the distribution, population structure, and evolutionary direction of germplasm resources of wild plants [41, 42]. Zhao et al. [26] divided cultivated tea plants in the Guizhou Plateau into four groups with reference to the river basin distribution. Our results showed that 159 wild tea plants from the Guizhou Plateau were divided into three groups—two pure groups (GP01 and GP02) and one mixed group (GP03)—and this grouping was consistent with that of the PCA and phylogenetic analysis.

Geological heterogeneity is known to impact species diversity [43]. The mineral content of soil is instrumental in the genetic differentiation of species populations [44]. In this study, the GP01 population was III H3 *C. gymnogyn* distributed in the SR Classes, while the GP02 and GP03 populations were II H1 *C. tachangensis* and I H2 *C. tachangensis*, respectively, distributed in the CR Classes. CR soil is rich in calcium and has a high content of organic matter (calcium-humus), including soil TN, AN, TP, AK and exchangeable Mg, while SR soil is rich in AP and exchangeable Al [45]. We observed higher gene flow, lower genetic distance and genetic differentiation between II H1 *C. tachangensis* and I H2 *C. tachangensis*, while the lowest gene flow and the highest genetic distance and genetic differentiation were observed between III H3 *C. gymnogyn* and II H1 *C. tachangensis*. Therefore, III H3 *C. gymnogyn* and II H1 *C. tachangensis* have the least gene exchange, and they assume different evolutionary directions due to different soil nutrient contents. With the spread of wild tea plants in the Guizhou Plateau, the primitive II H1 *C. tachangensis* gradually differentiated into III H3 *C. gymnogyn* in the process of transmission from CR to SR, so as to better adapt to the lower soil nutrients and meet the demand for P and Al. It indicated that rock types and soil nutrients developed from rock played an important role in the genetic differentiation of *C. tachangensis* and *C. gymnogyn*.

The pH values of soil developed from different rocks are distinct. The pH of soil developed from rocks with high calcium and magnesium contents was higher than that from rocks with high aluminum content [46, 47]. Moreover, the pH of soil from dolomitic limestone, dolomite, and clastic rock gradually decreased [48]. In this study, the II H1 *C. tachangensis* population was predominantly distributed in area II of the CR Classes, the I H2 *C. tachangensis* population was mainly distributed in area I of the CR Classes, and the III H3 *C. gymnogyn* population was predominantly distributed in area III of the SR Classes. The II H1 *C. tachangensis* were relatively primitive tea plants. In this study, the II H1 *C. tachangensis* growing area II has evolved into I H2 *C. tachangensis* growing area I, and then evolved into the H3 III *C. gymnogyn* growing area III in the process of communication by spreading. The II H1 *C. tachangensis* were growing in relatively higher pH soil from dolomitic limestone, the I H2 *C. tachangensis* were growing in relatively medium pH soil developed from dolomite, and the III H3 *C. gymnogyn* were growing in relatively lower pH soil from clastic rock. Ca and Mg ions alkalize soil, while Al ions acidify soil [47]. Our observations suggest that high pH II H1 *C. tachangensis* and medium pH I H2 *C. tachangensis* have a moderate of differentiation, while high pH II H1 *C. tachangensis* and low pH III H3 *C. gymnogyn* have a higher of genetic differentiation. Soil pH plays an important role in the propagation and differentiation of wild tea plants in the Guizhou Plateau and maintains the different evolutionary directions of the plant populations.

The II H1 *C. tachangensis* populations were mainly distributed at higher altitude with low temperature, the I H2 *C. tachangensis* populations were predominantly distributed at middle altitudes with high temperature, and the III H3 *C. gymnogyn* populations were mainly distributed at lower altitudes with high temperature. We observed the highest gene flow and the lowest genetic distance and genetic differentiation between III H3 *C. gymnogyn* and I H2 *C. tachangensis*, higher gene flow and lower genetic distance and genetic differentiation between II H1 *C. tachangensis* and I H2 *C. tachangensis*, and the lowest gene flow and the highest genetic distance and genetic differentiation between III H3 *C. gymnogyn* and II H1 *C. tachangensis*. Thus, the genetic differentiation between III H3 *C. gymnogyn* and II H1 *C. tachangensis* is the largest. Due to the difference of temperature at different altitudes, the genetic differentiation between *C. tachangensis* and *C. gymnogyn* was caused, which was congruent with previous studies [49–51]. Loricaria populations will differentiate from high to low altitude to form distinct genetic populations [52]. In this study, II H1 *C. tachangensis* distributed in higher altitude areas gradually spread to middle altitude areas, and then evolved into I H2 *C. tachangensis* under the adaptation of suitable temperature and soil rich in soil nutrients developed by dolomite. Subsequently, continued spreading of the I H2 *C. tachangensis* to the lower altitude resulted in their evolution into III H3 *C. gymnogyn* by natural selection on the clastic rock most suitable area to meet the demand for Al. We detected the highest and lowest genetic diversity in I H2 *C. tachangensis* and II H1 *C. tachangensis,* respectively. It is possible that the I area at medium altitude may be due to the high content of soil nutrients, suitable temperature, and frequent genetic exchanges among wild tea plants, which have increased their genetic diversity. In contrast, the II area at higher altitude may be suitable for local adaptation of tea plants owing to the lower temperature and rich soil nutrients, thereby reducing genetic diversity of the wild tea plants.

Wang [53] believed that most of the high frequency alleles of the plants bred outside (such as cross-pollinated wind-borne plants) appeared in each population, with high similarity, less differences, and small genetic variation among populations. Our results demonstrate that most of the genetic variation for wild tea plants exists within populations (91.5%) and not among populations (8.5%), which is consistent with previous reports [54, 55]. Tea plant is a woody plant with cross-pollination and a long life. Through continuous cross-pollination, tea plants distributed in the Guizhou Plateau have repeatedly carried out gene exchange between different species and populations, resulting in small differences between populations. This may verify the hypothesis that tea plants in Guizhou Plateau originated from the same species.

## Conclusions

The highest genetic diversity was observed for the I H2 *C. tachangensis* group (GP03) growing in I areas at middle altitude, while the II H1 *C. tachangensis* group (GP02) growing in the II areas at high altitude had the lowest genetic diversity. In the Guizhou Plateau, the suitable temperature in the middle altitude and the higher nutrient content of soil developed from dolomite promote the formation of high genetic diversity, while the low temperature at high altitude and the high pH of soil developed from limestone with dolomite promote the formation of low genetic diversity. Temperature and, soil nutrients and pH are the main factors affecting the genetic diversity of wild tea plants in the Guizhou Plateau. Findings from this study will facilitate understanding of the adaptive evolutionary characteristics of wild tea plants in the Guizhou Plateau, and will provide reference suggestions for further research on wild tea germplasm resources.

## Methods

### Plant materials

In total, 159 wild tea germplasm were used in this study (Additional file 1: Table S1). Of these, 101 were identified as *Camellia tachangensis* (F.C.Zhang) and 58 were *Camellia gymnogyna* Chang according to the classification system reported by Chen [34] and Min [56]. Based on the combined information of the agricultural climate zoning map of Guizhou Plateau, the suitable tea-planting areas in Guizhou Plateau, and the field survey growth altitude of wild tea plants [57, 58], the altitude of the wild tea-growing areas in Guizhou was divided into three gradients: > 1400 m was divided into the first altitude gradient (H1), 1400–1100 m was the second altitude gradient (H2), and < 1100 m was the third altitude gradient (H3). Among the 159 materials in the study, 39 were located at H1, 65 were located at H2, and 55 were located at H3 (Additional file 2: Fig S1). Based on the stratigraphic regionalization and the development history of the geological environment, the rock type of Guizhou Plateau where tea plants grew was divided into the Carbonate Rock (CR) and Silicate Rock (SR) Classes (Additional file 1: Table S1) [3]. The CR Class contained the Dolomite sub-suitable area (I) and the Dolomitic limestone suitable area (II), while the SR Classes contained the Clastic rock most suitable area (III) and the Purple clastic rock suitable area (IV). Among the 159 tea accessions, 92 were distributed in the CR Classes, including 48 samples from a Dolomite sub-suitable area (I) and 44 samples from a Dolomitic limestone suitable area (II). There were 67 tea accessions in the SR Class, of which 46 samples were from a Clastic rock most suitable area (III) and 21 samples were from a Purple clastic rock suitable area (IV).

Meteorological data of the distribution areas of wild tea germplasm resources were collected from the meteorological departments of all cities and counties in Guizhou Province. Specifically, these data comprised that from meteorological observation stations of all towns and townships, historical meteorological data recorded in the city annals, and the average values of the mean annual temperature (MAT) and mean annual rainfall (MAR) over 5 years (Additional file 1: Table S7). Geographical and altitude information were acquired with GPS, and rock types were judged according to a lithological map of Guizhou Province (Additional file 1: Table S1). The altitude data of Guizhou Plateau was from the geospatial data cloud (http://www.gscloud.cn/sources/index?pid=1&rootid=1) and is mapped using GIS techniques. Professor Suzhen Niu from the Key Laboratory of Plant Resources Conservation and Germplasm Innovation in Mountainous Region, Guizhou University, Ministry of Education, Institute of Agro-Bioengineering, associate professor Jie Yin and lecturer Qinfei Song of the College of Tea Science of Guizhou University, and researcher Zhengwu Chen of the Institute of Tea Science, Guizhou Academy of Agricultural Sciences conducted morphological identification on all sampled tea plants, and stored them in the tea germplasm resource garden of Guizhou University (Additional file 3). This field study and experimental research complied with local legislation, and national and international guidelines. The authors also complied with the Convention on the Trade in Endangered Species of Wild Fauna and Flora and Regulations of Guizhou Province on the protection of ancient tea plants.

### DNA extraction, library construction, and sequencing

Genomic DNA was extracted from tea plants using the Rapid Extraction Kit of Plant Genomic DNA (Beijing Biomed Gene Technology Co. Ltd., Beijing, China) according to the manufacturer’s instructions. The genomic DNA was digested with the restriction endonucleases SacI and MseI (5 U; New England Biological Laboratory (NEB), Ipswich, USA), then the adapters “SacAD and MseAD”, which have unique barcodes, were ligated to the DNA fragments. Fragments of 500-–550 bp were selected for amplification and sequenced on an Illumina Hi Seq platform (Illumina, San Diego, CA, USA). The length of the original paired-end sequence is 150 bp [13, 59].

### Sequence alignment and SNP identification

Barcodes were used to multiplex raw DNA reads, and the adapters were removed using custom Perl scripts. Only 5 read quality values > were retained. The reads were mapped to the reference genome (http://tpia.TeaPlant.org/) using BWA-MEM v. 0.7.10 (https://sourceforge.net/projects/bio-bwa/files/) with default parameters [26]. SNPs were filtered according to the methods of Niu et al. [13] and McKenna et al. [23]. The SNPs of MAF > 0.05 or the rate of missing data < 20% were saved with VCF tools v. 0.1.160 (https://github.com/vcftools/vcftools) [60]. The SNP density plot was drawn in CM plot v. 3.7.0 (https://rdrr.io/cran/CMplot/) [61]. A total of 98,241 SNPs were selected from 159 tea samples and subsequent analysis was conducted (Additional file 4: Table S1).

### Population structure and LD

ADMIXTURE v. 1.30 (http://dalexander.github.io/admixture/download.html) was used to speculate the proportions of admixtures among the wild tea populations, and the number of ancestries (k) was in the range of 1–9. The score threshold was set to 0.8 to distinguish between pure and admixture groups [61]. PCA was conducted in TASSEL v. 5.2.72 (https://tassel.bitbucket.io) [62]. The maximum likelihood (ML) phylogenetic tree was reconstructed by using MEGA v. 10.2.4 (https://www.megasoftware.net/dload_win_gui) to run 500 bootstrap replicates. The phylogenetic tree was displayed by iTOL [63]. Using PopLDdecay v. 3.29 (https://github.com/BGI-shenzhen/PopLDdecay) with default parameters, the LD was calculated statistically based on the correlation coefficient (R^2^) of the unpruned pairwise SNPs throughout the genome [64]. The VCF file was converted to a pedigree file using VCFtools v. 0.1.160 [64].

### Genetic diversity

The genetic diversity indexes included *Ho*, *Pi*, *MAF*, *Fis*, and Tajima’s D. *Ho*, *MAF*, and *Fis* of each inferred population were calculated using Plink v. 1.90 (https://www.cog-genomics.org/plink2/) [65]. VCFtools was employed to calculate *Pi* and Tajima’s D of each inferred population and *Fst* of the paired inferred population [60]. *Fst* was then used to calculate *Nm* according to the formula *Nm* = (1 − *Fst*)/4*Fst* [26]. MEGA v. 10.2.4 was used to calculate *GD* for the pairwise inferred populations. Significant differences between these indexes and the single factor analysis of variance (ANOVA) of soil nutrients in different groups were calculated using SPSS v.25 (IBM Corp., Armonk, NY, USA) [66]. Analysis of molecular variance (AMOVA [67]) was conducted in Arlequin v.2.000 [68] to determine the division of the overall genetic variation at two levels: within the population and between species.

### Soil chemical characteristics

In the distribution area of wild tea germplasm resources, select the area with the largest population density of wild tea plants and set it at 600m^2^ (20 m × 30 m). Next, five points were set on the diagonal of each sample plot, and one soil profile was dug at each point by removing 0–5 cm dead leaves from the surface layer and then collecting 10–60 cm of the soil layer. The soil samples collected at the five points were mixed and impurities and stones were removed. Approximately 1 kg of soil samples were subjected to the quartering method, spread on white paper indoors, and allowed to dry, naturally, then plant roots and stones were picked out. The resulting samples were ground with wooden sticks and passed through a nylon sieve [21]. The soil samples were analyzed for soil organic matter (SOM), total nitrogen (TN), available nitrogen (AN), total phosphorus (TP), available phosphorus (AP), available potassium (AK), exchangeable Ca, exchangeable Al, exchangeable Mg, and pH based on standard methods (Additional file 1: Table S7) [69]. Briefly, SOM was determined by the wet combustion process of potassium dichromate. TN and AN were analyzed by the Kjeldahl method and basic nitrogen diffusion method respectively. TP was determined by the HClO_4_-H_2_SO_4_ method. AK and AP were determined by flame emission spectrometry and the Olsen method, respectively. Exchangeable Ca and exchangeable Mg were measured by continuous source atomic absorption spectrometry. The pH of the soil was tested with a glass electrode (ratio of soil to water = 1:2.5).

## Supplementary Information

**Additional file 1: Table S1.** Information of 159 wild tea accessions used in this study. **Table S2.** The quality control data of 159 wild tea accessions. **Table S3.** Statistics of Heterozygosity Rate of 98,241 SNPs in 159 wild tea accessions. **Table S4.** SNP density. **Table S5.** Statistics of the number and ratio of the accessions of species, rock classes and geologically suitable area in three inferred populations. **Table S6.** Statistics of the number and ratio of the accessions of altitude gradient in three inferred populations. **Table S7.** The soil nutrient content and altitude factor of 159 wild tea accessions.

**Additional file 2: Figure S1.** Geographic distribution of 159 materials collected at different altitudes and rock types. Note: (A) Geographical position. (B) Distribution map of altitude, rock type and sample point in Guizhou Plateau. **Figure S2.** Graph for CV error in the range of K=1-9 of 159 wild tea accessions. **Figure S3.** ML tree of four geologically suitable areas. Note: I Dolomite sub-suitable area, II Dolomitic limestone suitable area, III Clastic rock most suitable area, IV Purple clastic rock suitable area.

**Additional file 3: Table S1.** Sampling localities of 159 wild tea accessions.

**Additional file 4: Table S1.** Genotyping of 98,241 SNPs based on GBS in 159 wild tea accessions.

## Data Availability

The plant materials are growing in our resource nursery and are available from the corresponding author on reasonable request. The raw sequence data reported in this study have been deposited in the Genome Sequence Archive [70] in BIG Data Center, Beijing Institute of Genomics (BIG), Chinese Academy of Sciences, under accession number CRA001438, and are publicly accessible at http://bigd.big.ac.cn/gsa.

## Abbreviations

CV error: Cross-validation error
Fis: Inbreeding coefficient
Fst: Differentiation coefficients
GBS: Genotyping-by-sequencing
GD: Genetic distance
GDR: Genetic distance range
GWAS: Genome-wide association studies
Ho: Observed heterozygosity
LD: Linkage disequilibrium
MAF: Minor allele frequency
ML tree: Maximum-Likelihood tree
Nm: Gene flow
PCA: Principal component analyses
*Pi*: Nucleotide diversity
SNPs: Nucleotide polymorphisms
*C.Tachangensis*: *Camellia **tachangensis* (F.C.Zhang)
*C. **Gymnogyna*: *Camellia **gymnogyna* Chang
CR: Carbonate Rock
SR: Silicate Rock
