# Supplementary material for: Population structure analysis to explore genetic diversity and geographical distribution characteristics of wild tea plant in Guizhou Plateau
Source: BMC Plant Biol. 2023 May 16;23:255. doi: 10.1186/s12870-023-04239-2 (PMC10186670; doi:10.1186/s12870-023-04239-2)
Supplement: Supplementary file 2 — Additional file 2: Figure S1. Geographic distribution of 159 materials collected at different altitudes and rock types. Note: (A) Geographical position. (B) Distribution map of altitude, rock type and sample point in Guizhou Plateau. Figure S2. Graph for CV error in the range of K=1-9 of 159 wild tea accessions. Figure S3. ML tree of four geologically suitable areas. Note: I Dolomite sub-suitable area, II Dolomitic limestone suitable area, III Clastic rock most suitable area, IV Purple clastic rock suitable area. [file 12870_2023_4239_MOESM2_ESM.docx]

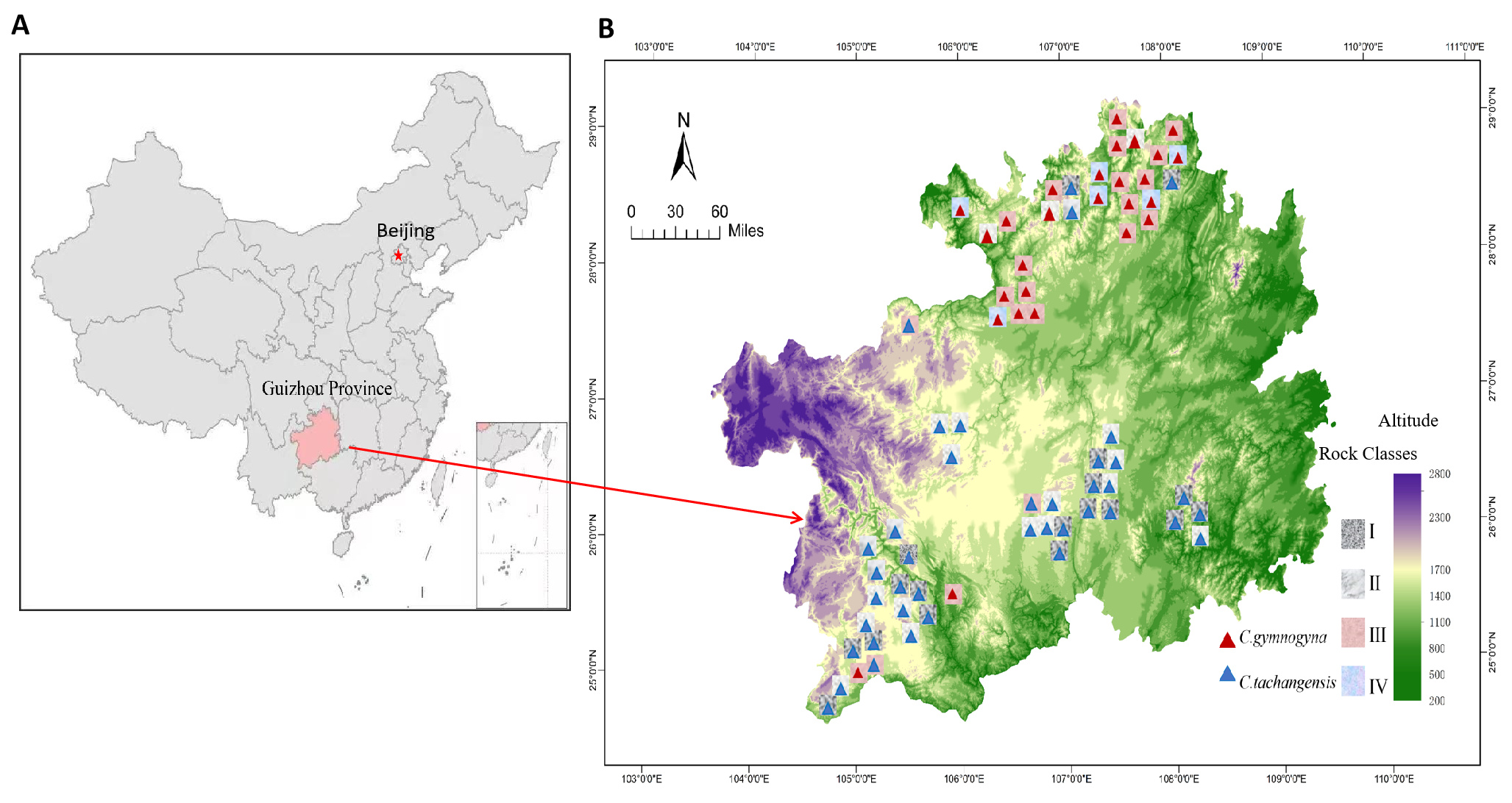


**Fig. S1 Geographic distribution of 159 materials collected at different altitudes and rock types.**

Note: (A) Geographical position. (B) Distribution map of altitude, rock type and sample point in Guizhou Plateau


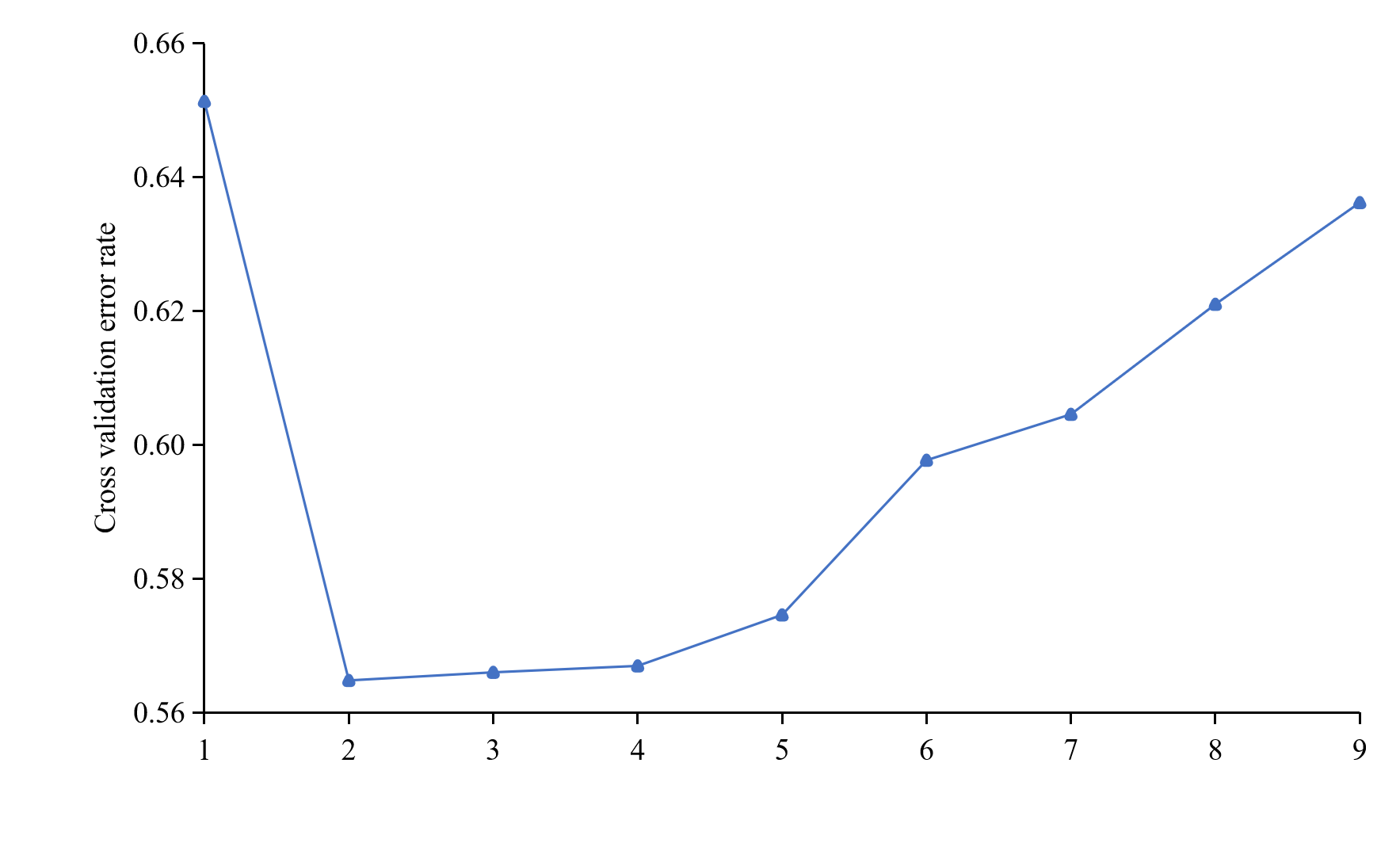


**Fig. S2 Graph for CV error in the range of K=1-9 of 159 wild tea accessions.**


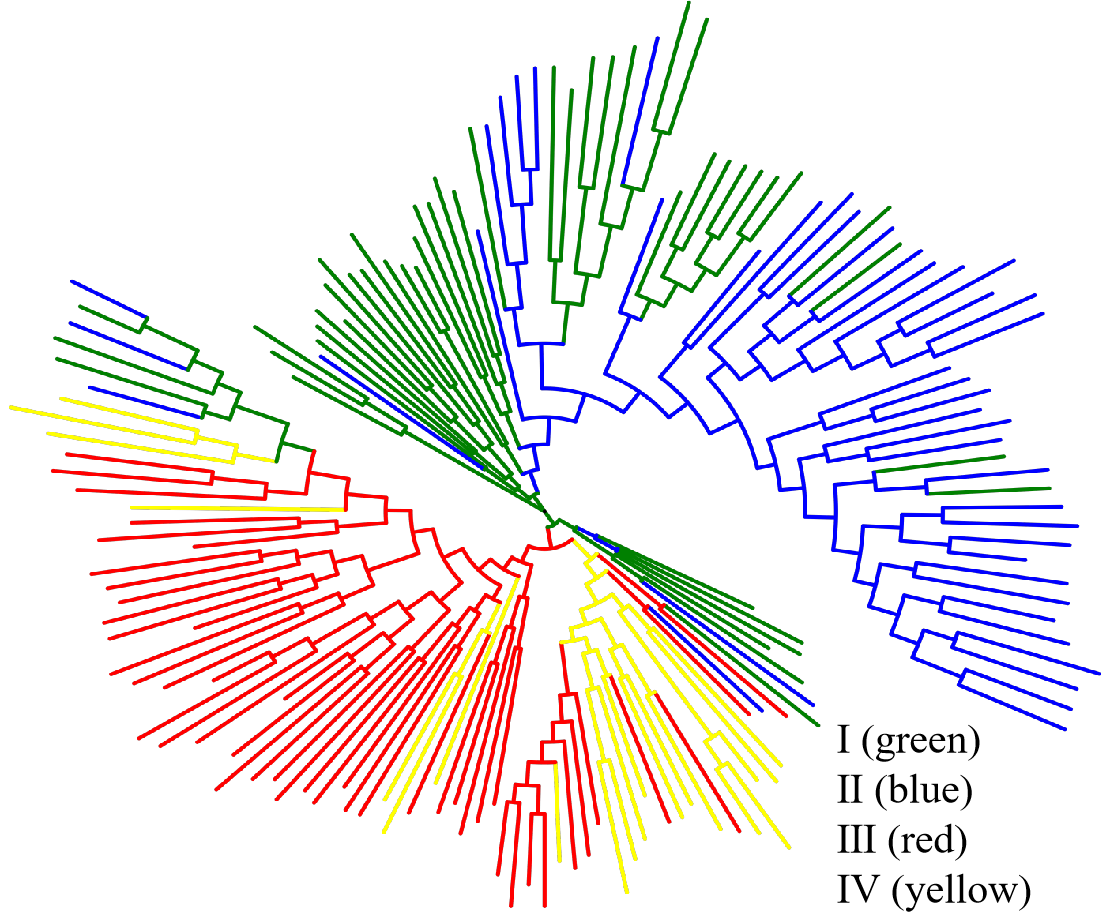


**Fig. S3 ML tree of four geologically suitable areas.**

Note: I Dolomite sub-suitable area, II Dolomitic limestone suitable area, III Clastic rock most suitable area, IV Purple clastic rock suitable area
